# Case Report: Psychogenic purpura in a uremic patient on peritoneal dialysis

**DOI:** 10.3389/fimmu.2025.1625126

**Published:** 2025-09-12

**Authors:** Lin Zhang, Hanqing Zhang, Yuetong Zhao, Tao Zhang, Zhengjie Zhu, Yanheng Qiao, Yongming Tian, Hang Su, Jie Li, Bo Yang

**Affiliations:** Department of Nephrology, First Teaching Hospital of Tianjin University of Traditional Chinese Medicine, National Clinical Research Center for Chinese Medicine Acupuncture and Moxibustion, Tianjin, China

**Keywords:** psychogenic purpura, gardner-diamond syndrome, autoerythrocyte sensitization syndrome, uremia, autoimmunity, differential diagnosis

## Abstract

Psychogenic purpura (Gardner-Diamond syndrome) is a rare autoimmune vasculopathy characterized by the spontaneous onset of painful edema and infiltrative cutaneous lesions that rapidly develop into ecchymosis after severe psychological stress events. In this article, we report an 87-year-old female uremic patient who was admitted to the hospital with erythema and subcutaneous ecchymoses on the head and face following an Aedes mosquito sting. She was previously diagnosed with “toxic insect stings and skin bacterial infections” and was given anti-infective treatment by an outside hospital, which was ineffective. Subsequent laboratory tests at our hospital revealed only an increase in fibrinogen and leukocytosis. Tracing the history revealed that the patient’s purpura episodes were related to a major life event, the death of her husband. After consultation with the dermatology department, the patient’s autoerythrocyte sensitization test was positive, and she was finally diagnosed with “psychogenic purpura”. Treatment included glucocorticoids and immunomodulators, supplemented by anti-infective and renal replacement therapy, and the patient’s ecchymosis gradually subsided and resolved after one month of follow-up. This case highlights the complexity of diagnosing psychogenic purpura and the significance of medical history in the diagnosis. Only accurate and timely diagnosis can effectively avoid unnecessary treatment.

## 1 Introduction

Psychogenic purpura (PP), also known as Gardner-Diamond syndrome, autoerythrocyte sensitization syndrome, or painful bruising syndrome, is a rare disease. To date, only more than 280 cases have been reported worldwide (1). It was first described by Gardner and Diamond in 1955 in the U.S (2). PP primarily affects young to middle-aged women under the age of 50, accounting for more than 90% of cases (3). Patients usually have a history of mental disorders or have experienced extreme psychologically distressing events (4, 5). It is characterized by painful edema and infiltrative skin lesions induced by psychological stress, which subsequently develop into ecchymosis within 24 hours and expand into larger ecchymosis within 4 to 5 days. Other typical symptoms include gastrointestinal symptoms, arthralgia, myalgia, dizziness, and in some cases, hematuria and oral bleeding (6–8). The pathogenesis of PP is unclear, with studies suggesting that it may involve auto-sensitization to erythrocyte membrane phosphoglycerol esters, and others suggesting a link to oestrogens or hypovolemia (3).

The diagnosis of PP is challenging, as its clinical manifestations often resemble infections, coagulation disorders, or autoimmune disorders, making it easy to misdiagnose it as dengue, idiopathic thrombocytopenic purpura (ITP), or systemic lupus erythematosus (SLE). Due to the lack of a gold standard for diagnosis, PP is usually a diagnosis of exclusion. A correct diagnosis can only be made after a thorough medical history taking and laboratory examination. In this article, we report a case of PP in an elderly Chinese female uremic patient who got it after the death of her husband and an Aedes mosquito sting, and discuss its clinical features and the difficult diagnostic process.

## 2 Case presentation

**Figure 1:**
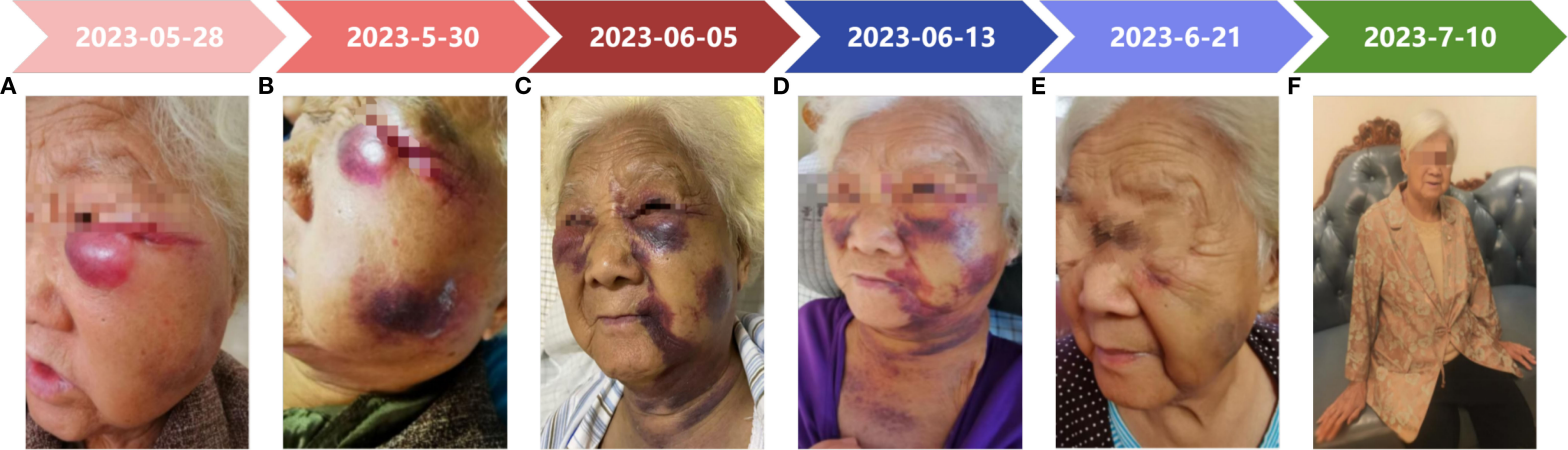
A timeline of the patient’s symptom progression. **(A, B)** The patient presented with redness, swelling, and pain on the left cheek, with a palpable 2×2 cm subcutaneous hard nodule. Multiple ecchymoses were noted on the head and neck. **(C)** The patient’s left cheek exhibited a darkening of the induration, with ecchymoses spreading to the anterior chest. **(D, E)** The patient’s ecchymoses had lightened in color and were gradually fading. **(F)** The patient had recovered.

**Figure 2:**
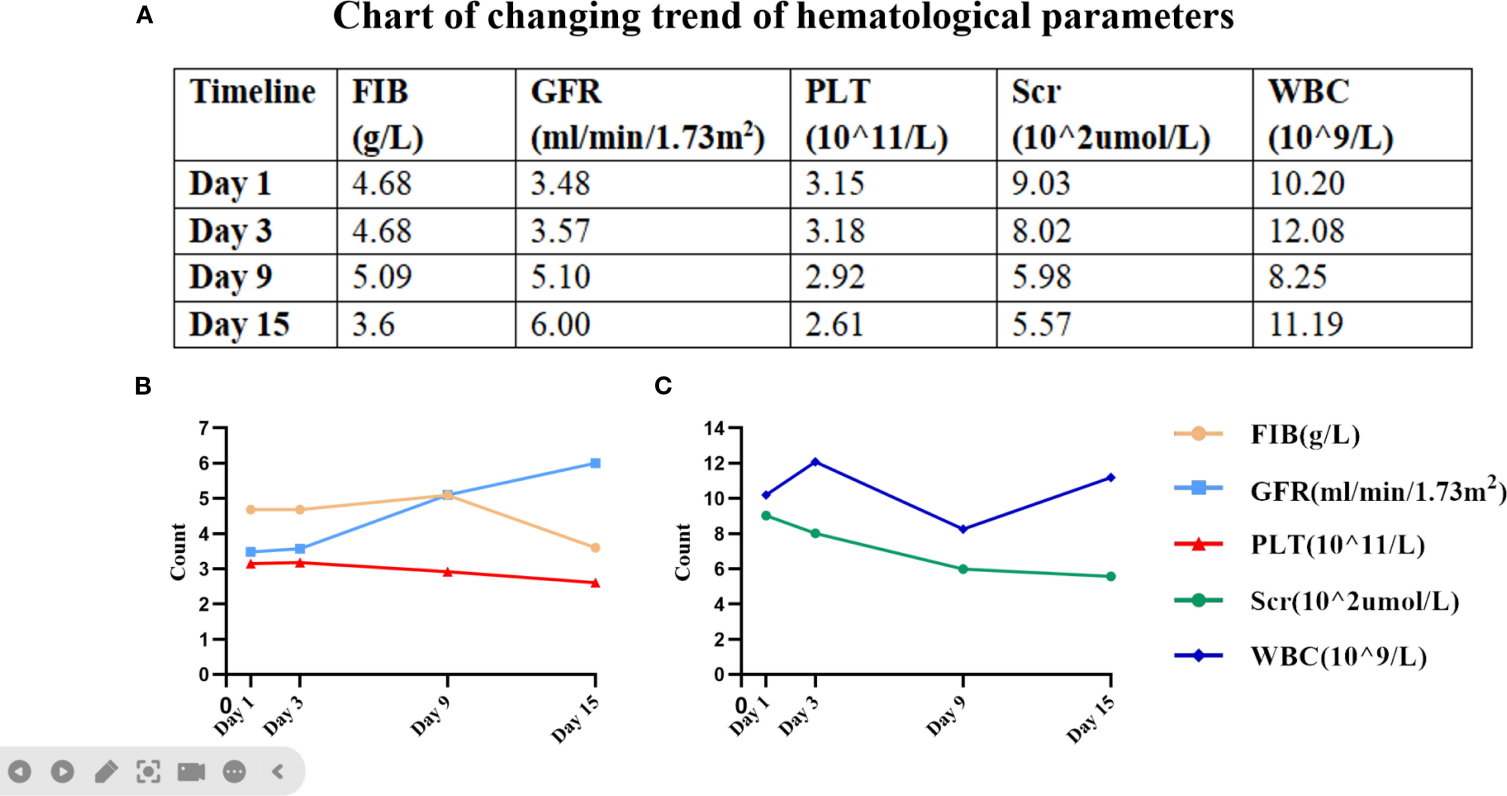
Key hematologic parameters from admission to discharge. **(A)** A graph of specific values corresponding to the figure. **(B)** The trends in fibrinogen (FIB), glomerular filtration rate (GFR) and platelet count (PLT). **(C)** The trends in blood creatinine (Scr) and white blood cell (WBC) count.

On May 30, 2023, an 87-year-old woman was admitted to the hospital with a 2-day history of redness and swelling of the head and face after an Aedes mosquito sting, as well as multiple painful ecchymoses on the head, face and neck. She had a history of hypertension and chronic renal failure. With regular treatment, the condition was stable. She denied any other chronic diseases, infectious diseases and allergies. After being stung by an Aedes mosquito on the left cheek during outdoor activities 2 days ago, the patient rapidly developed redness, swelling and pain on the left cheek, with a palpable 2×2 cm subcutaneous hard nodule, followed by extensive subcutaneous ecchymoses on the head and neck, which were obvious to palpation (**Figure 1A**). At that time, she was examined in a foreign hospital, which indicated the following: coagulation function: fibrinogen (FIB) 4.68 g/L, bleeding time (BT), prothrombin time (PT) and partial thromboplastin time (PTT) were normal; renal function: see **Figure 2**; blood routine: white blood cell count (WBC) 10.20×10^9^/L (93.8% neutrophils), hemoglobin (Hb) 107g/L, and the rest of the indicators were roughly normal; inflammatory indicators: serum amyloid A 13.21 mg/L; maxillofacial MR: infectious lesions with abscess formation in the left temporal, maxillofacial, and cervical regions, and chronic inflammation of the sieve sinuses and maxillary sinuses bilaterally. Based on the patient’s normal BT, PT, PTT, and PLT results, ITP, factor XIII deficiency, and other coagulation disorders were excluded. She was diagnosed with chronic renal failure (uremic stage), toxic insect stings, and skin bacterial infections, and was referred to our hospital because of the ineffectiveness of antibiotic treatments.

On admission, the patient’s vital signs were normal; physical examination revealed redness, swelling, and pain in the left cheek, with a palpable subcutaneous hard nodule of 2×2 cm, and multiple subcutaneous ecchymoses in the head and neck (**Figure 1B**). Further refinement of laboratory tests showed that coagulation function, blood routine indexes and renal function were not significantly different from before (**Figure 2**); electrolyte: calcium 2.04 mmol/L, phosphorus 1.53 mmol/L; inflammation indexes: procalcitonin 0.21 ng/ml; stool routine: OB (+). The preliminary diagnosis was “chronic renal failure (uremia stage), renal anemia, renal bone disease, hyperphosphatemia, hypertension grade 3, gastrointestinal hemorrhage, toxic insect stings, skin bacterial infections, dengue”, and the treatment was based on symptomatic therapy such as regular peritoneal dialysis, antibiotic treatments, supplementation of protein raw materials, lowering of phosphorus, lowering of blood pressure, and protection of gastrointestinal mucosa. Specific medications are as follows: 2000ml of 1.5% peritoneal dialysis solution three times a day, intravenous piperacillin sodium and tazobactam sodium 3.375g every 8 hours, oral compound α-keto acid 1.89g/day, oral sevelamer carbonate 1.6g/day, oral amlodipine besylate 5mg/day, and intravenous omeprazole sodium 40mg/day.

**Figure 3:**
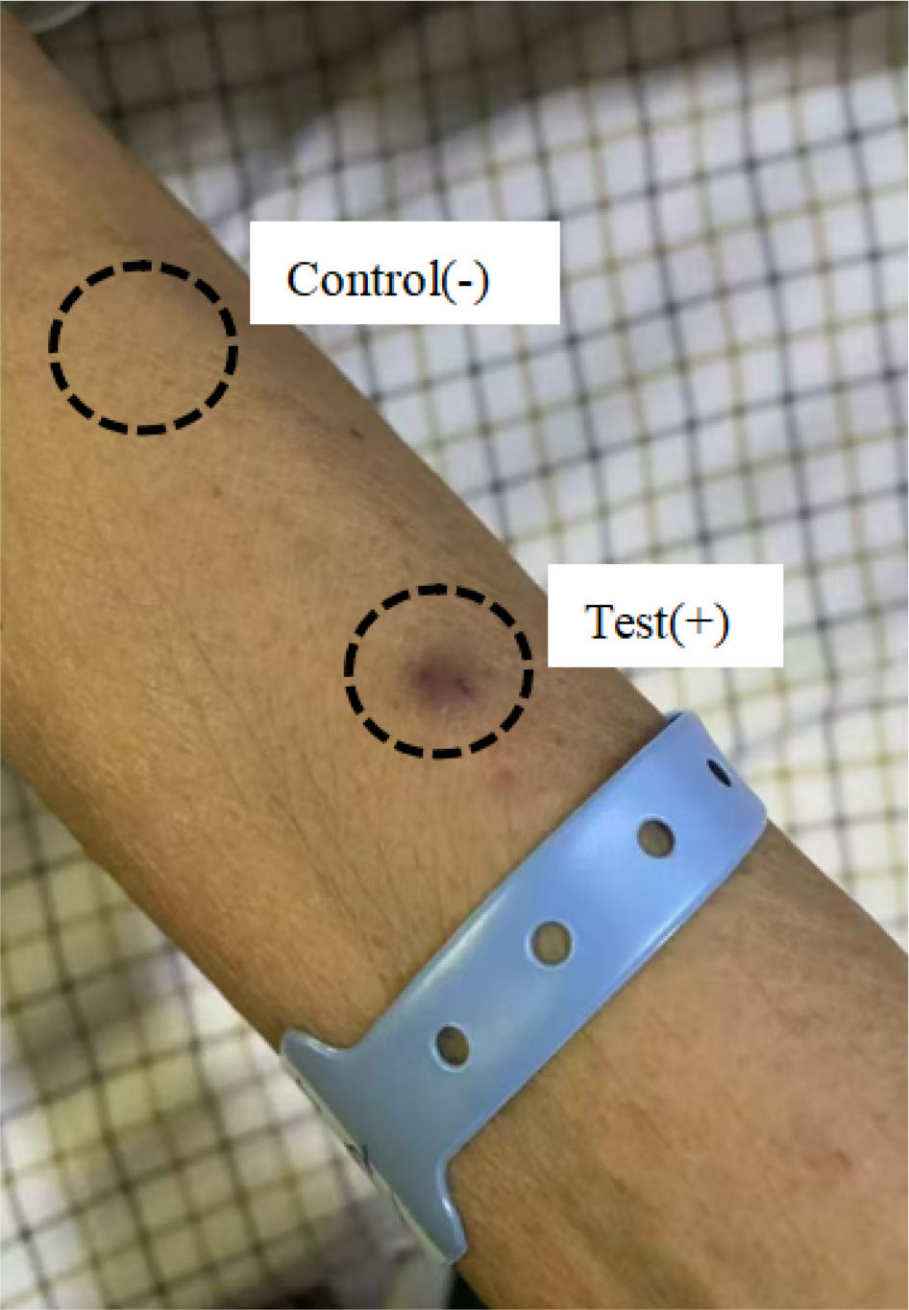
An autoerythrocyte sensitization test: positive with an ecchymosis, negative with saline control.

On May 31, 2023, a dermatology consultation was requested, and the immune-related examinations showed weakly positive antinuclear antibody (ANA), IgG 6.620 g/L, hemosiderosis (ESR) 65.0 mm/h, and C-reactive protein (CRP) 99.500 mg/L. Other indicators such as anti-streptococcal hemolysin “O” (ASO), anti-double-stranded DNA (anti-dsDNA) and rheumatoid factor (RF) were all negative, according to which we excluded the diagnosis of dengue and other immune-related diseases. In the course of the ward rounds, we asked the patient and her family about her medical history in detail again. The family complained that two days before admission, the patient was in grief because of her husband’s burial, and she suddenly suffered from chest tightness and breathlessness. Then she was sent to the hospital by ambulance and was in a coma during the emergency transportation due to excessive grief. Based on this important history of psychological stress, combined with the patient’s symptoms and laboratory indicators, the dermatology department diagnosed “psychogenic purpura, skin bacterial infections”, and the patient’s right forearm was sensitized to auto-erythrocytes. The dermatologist recommended anti-inflammatory, immunomodulatory, and antibiotic treatments, including immediate intravenous dexamethasone sodium phosphate 3 mg, oral prednisone acetate 12 mg/day, oral thalidomide 50 mg/day, intravenous vitamin C 2 g/day, oral rutin 120 mg/day, and intravenous antibiotics as before. After 24 hours, the autoerythrocyte sensitization test was positive, with a burning sensation and ecchymosis; meanwhile, the saline control was negative, confirming the diagnosis of PP (**Figure 3**).

On June 5, 2023, the patient’s hard nodule on the left cheek darkened, pressure pain decreased, and ecchymoses spread to the anterior chest (**Figure 1C**). Continuous monitoring of blood indicators (**Figure 2**) revealed a lower white blood cell count than before, suggesting the effectiveness of antibiotic treatments. The patient’s symptoms improved, so the medication regimen was adjusted: oral prednisone acetate and intravenous vitamin C were discontinued, and oral methylprednisolone 8 mg/day was initiated.

On June 13, 2023, the patient’s ecchymoses on the head and neck became lighter in color and gradually dissipated without pressure pain (**Figure 1D**). Reviewing the blood indices (**Figure 2**), the patient’s white blood cell count was elevated, but only the monocyte count (1.73×10^9^/L) was elevated. A repeat thin-layer CT of the maxilla showed that the subcutaneous soft tissue of the left cheek presented as nodular, slightly hyperdense shadows, accompanied by a slight swelling, and the inflammation of the bilateral maxillary sinuses had lessened than before. The patient’s symptoms improved without fever and other discomforts, so the raised white blood cells were attributed to the use of immunomodulators, and the immunomodulators were stopped after 2 days.

**Figure 4:**
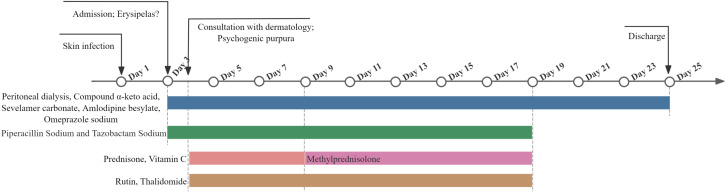
A timeline of diagnostic and treatment.

On June 21, 2023, the patient was discharged from the hospital with significant improvement (**Figure 1E**), and a review of the diagnostic and treatment timeline is shown in **Figure 4**. On July 10, 2023, we followed up with the patient’s family by telephone, who indicated that the patient’s psychogenic purpura had resolved (**Figure 1F**). Currently, the patient has been followed for 1.5 years with no signs of recurrence.

## 3 Discussion

PP is a rare bleeding disorder of the skin, usually induced by psychological stress. The onset of the disease is often preceded by physiological stressors, such as minor mechanical injury, stress, surgery, or hard physical labor, as well as prodromal symptoms, such as pain, burning sensation, and itching. The disease is characterized by painful edema and infiltrative lesions of the skin, which subsequently develop into ecchymosis within 24 hours, and expand into larger ecchymosis within an average of 4 to 5 days. Skin changes may be associated with a variety of systemic diseases, including fever, arthralgia, myalgia, headache, and dizziness. More than half of the PP patients reported different gastrointestinal symptoms, including epigastric pain, gastrointestinal bleeding, nausea, vomiting, and diarrhea. In addition, some studies have documented symptoms of hematuria and oral bleeding (6–8).

The pathogenesis of PP has not been fully elucidated. In 1955, Gardner and Diamond suggested that the disease may involve auto-sensitization of erythrocyte membrane phosphoglycerides (2). In 1968, Ratnoff and Agle first proposed a psychosomatic basis for PP, emphasizing that there is a direct relationship between mental stress and cutaneous manifestations, which gave birth to the name “psychosomatic purpura” (9). Currently, the pathogenesis of PP is considered to be a synergistic interaction between psychoimmunology and mechanical damage to the skin, in which preexisting psychosomatic disorders may affect skin immune function and weaken dermal capillaries. As a result, even minor skin injuries may disturb the capillary walls, ultimately leading to erythrocyte infiltration (10). Based on the pathogenesis of PP, the autoerythrocyte sensitization test is considered a criterion to confirm the diagnosis. The test is performed by injecting self-washed erythrocytes into the flexor surface of the forearm, and the results are usually read after 30 minutes and 24 hours, with the presence of induration and ecchymosis at the injection site deemed positive (11). However, some patients with PP have a negative response to this test, and a previous review showed a positive test rate of 85.7% (12). Therefore, this test can only be used as a reference, and not as a gold standard for diagnosing PP.

The diagnosis of PP presents significant complications. First, its clinical presentation lacks specificity and needs to be differentiated from dermatitis artefacta (DA), ITP, factor XIII deficiency, anaphylactic purpura, vascular hemophilia, Pfeifer-Weber-Christian syndrome, Ehlers-Danlos syndrome, malignant hematological disorders, dengue, vasculitis, and SLE (13). If necessary, the possibility of domestic violence and abuse should also be considered (14). Most of the above diseases have specific laboratory indicators and are not difficult to distinguish clinically. However, since DA and PP often show no obvious abnormalities in laboratory tests, and both are more common in women and accompanied by psychiatric symptoms, differential diagnosis becomes more challenging. At this point, a detailed medical history is crucial. DA patients typically have a history of self-harm, often intentionally damaging their skin through mechanical or chemical means, resulting in diverse skin lesions such as erythema, blisters, erosion, and crusting (15). In contrast, PP patients do not exhibit self-harm behaviors, and their skin lesions are more uniform in appearance, typically developing into ecchymoses within 24 hours. Second, laboratory testings are typically unremarkable, with just a few patients showing modestly abnormal laboratory values that support the diagnosis of any hematologic or immunological disorder. Furthermore, the autoerythrocyte sensitization test lacks accuracy, which does not allow for complete confirmation of PP. Based on the above, the diagnosis of PP is often referred to as a “diagnosis by exclusion”. In diagnosing PP, the first step is to take a thorough medical history, looking for a history of mental diseases or psychological stressors, and looking for a correlation between psychological events and the onset of symptoms. Secondly, the clinical manifestations should be carefully observed to see if they conform to the typical evolutionary pattern of “physiological stressor → prodromal symptoms → painful edema → ecchymosis”, and to exclude diseases with similar manifestations by the accompanying symptoms. Finally, necessary laboratory tests should be performed, including CBC, ESR, PLT, BT, PT, PTT, CRP, direct and indirect Coombs test, ANA, anti-dsDNA, RF and complement level (16). In general, the absence of obvious abnormalities in each index proves that there is no underlying hematological disease or other recognizable pathology.

In this case, the patient had just experienced the extremely painful event of her husband’s death, and the onset of symptoms was highly synchronized with the psychological stressor. The clinical manifestations conformed to the typical evolutionary pattern of “physiological stressor → prodromal symptoms → painful edema → ecchymosis”, and the accompanying gastrointestinal bleeding was also a common concomitant symptom of PP. The diagnosis of PP was confirmed after thorough laboratory tests to rule out ITP, salpingitis, SLE, and others. Because the patient had concurrent skin infections and no platelet abnormalities, only infection-related diseases were initially considered, and the immune response caused by psychological factors was ignored. This emphasizes the need to have complete awareness of the patient’s medical history and consider the correlation between psychological status and disease occurrence when making a diagnosis.

In the treatment of PP, both psychological interventions and symptomatic support are usually administered (17). For psychological intervention, psychological counseling and social support are used, or antidepressants based on selective 5-hydroxytryptamine reuptake inhibitors (SSRIs). For symptomatic support, corticosteroids, antihistamines, and immunosuppressants are commonly used to relieve skin symptoms. The most recent systematic evaluation on PP showed that approximately 26% of patients relapsed after adequate follow-up, but only 8% of patients who received ≥1 psychiatric treatment (psychotherapy or medication) relapsed, resulting in 92% of patients in complete remission (3). Thus, psychological interventions are essential for achieving the best possible results.

In this case, the patient was only experiencing psychological stress due to the death of her husband, rather than a psychiatric disorder. Therefore, instead of prescribing antidepressants, we actively communicated with the family and instructed them to provide the patient with psychological comfort to ensure the patient received adequate social support. For symptomatic treatment, glucocorticoids and immunomodulators were used, among which dexamethasone, prednisone acetate and methylprednisolone could inhibit excessive inflammatory response, thalidomide reduced petechial inflammation by inhibiting inflammatory factors and angiogenesis, and rutin and vitamin C enhanced capillary stability in order to reduce erythrocyte extravasation. For concomitant symptoms, we applied antibiotics in sufficient quantity and course to treat skin infections caused by toxic insect stings, and used pantoprazole sodium for a long period to protect the gastrointestinal mucosa, thereby treating and preventing gastrointestinal bleeding. At the same time, we actively managed the primary disease by intensifying peritoneal dialysis to reduce edema pressure on the subcutaneous microcirculation, promote petechial absorption, and avoid further damage to the vascular endothelial barrier due to the accumulation of uremic toxins, such as indolephenol sulfate, caused by inadequate dialysis (18).

## 4 Conclusion

This patient is the second case of uremia combined with psychogenic purpura reported worldwide, following the first report in 2021 (19). This suggests that the possibility of psychogenic purpura should be considered when a uremic patient presents with painful ecchymosis. Diagnosis of PP requires complete awareness of the patient’s medical history, attention to the chronological association between psychological factors and clinical symptoms, and exhaustive laboratory tests to exclude other hematological and immunological disorders. Meanwhile, the successful management of this elderly uremic patient with PP confirms the effectiveness of a multidimensional treatment strategy. Through the comprehensive application of psychological intervention, immunomodulation, anti-infection and the treatment of primary disease, not only were the acute phase symptoms effectively controlled, but also the lasting effect of the treatment was verified by the long-term follow-up (ecchymoses completely disappeared after 1 month and did not recur for 1.5 years).

## Data Availability

The original contributions presented in the study are included in the article/supplementary material. Further inquiries can be directed to the corresponding authors.
